# Waldenström macroglobulinemia complicated with AL *λ* − type amyloid nephropathy: a case report and literature review

**DOI:** 10.3389/fmed.2026.1806896

**Published:** 2026-04-15

**Authors:** Yuan Ma, Taotao Li, Xiaoli Li

**Affiliations:** 1 Gansu University of Chinese Medicine, Lanzhou, China; 2 Department of Nephrology, Gansu Provincial Hospital, Lanzhou, China

**Keywords:** AL-type amyloidosis, IgM, MYD88-L265P mutation, nephrotic syndrome, prognostic analysis, Waldenström macroglobulinemia, *λ* light chain

## Abstract

Waldenström macroglobulinemia (WM) is a rare CD20-positive B-cell non-Hodgkin lymphoma. It is characterized by lymphoplasmacytic infiltration in the bone marrow and abnormal monoclonal IgM secretion. WM complicated by renal amyloidosis is uncommon but associated with rapid progression of organ damage. Accurate identification of pathogenic factors and individualized treatments are essential to improve prognosis. This paper reports the case of a 75-year-old female who initially presented with facial edema and bilateral lower-extremity edema lasting 2 days. Additional symptoms included foamy urine, frequent urination, and urgency. Laboratory tests revealed significant proteinuria (24-h urinary protein, 6.11 g), hypoalbuminemia (serum albumin, 20.07 g/L), and impaired renal function (serum creatinine, 129.90 μmol/L; eGFR, 45.2 mL/min/1.73 m^2^). Immunofixation electrophoresis detected monoclonal IgM-*λ* immunoglobulin in both serum and urine. Bone marrow biopsy demonstrated clonal lymphoplasmacytic infiltration (32%), and genetic testing revealed a positive MYD88-L265P mutation (mutation frequency, 28.5%). Renal biopsy indicated diffuse deposition of *λ* light chains predominantly in the glomerular mesangium. Ultrastructural examination revealed amyloid fibrils, confirming the diagnosis of AL *λ* − type amyloid nephropathy. Chemotherapy with rituximab, cyclophosphamide, and dexamethasone (RCD regimen), combined with symptomatic supportive therapy, resulted in temporary improvement of clinical symptoms and laboratory parameters. However, the disease progressed rapidly. The patient died on Jan 28, 2024, approximately 3 months after discharge, due to multiple organ failure involving cardiac, renal, and respiratory dysfunction. Based on relevant literature and the Chinese Expert Consensus on Diagnosis and Treatment of Lymphoplasmacytic Lymphoma (LPL)/WM (2022 Edition) [Abstract 3], this paper discusses diagnostic criteria, differentiation of pathogenic components, treatment strategies, and prognostic factors. These findings may provide clinical guidance for similar rare cases.

## 1 Introduction

Waldenström macroglobulinemia (WM) is the primary subtype of lymphoplasmacytic lymphoma (LPL), representing less than 2% of non-Hodgkin lymphomas (1, 2). The median age at diagnosis approximately 65 years. Its defining characteristics are lymphoplasmacytic infiltration in the bone marrow and abnormal secretion of monoclonal IgM paraprotein. The disease displays distinct molecular genetic features: MYD88-L265P mutation, with a rate exceeding 90%, is a critical diagnostic and prognostic marker. Additionally, CXCR4 mutations indicate greater disease aggressiveness (3).

Approximately 3% of patients with WM develop amyloidosis caused by the misfolding of abnormal proteins. The kidneys are the most commonly affected organs, primarily exhibiting AL-type amyloidosis (light chain-mediated), accounting for over 95% of cases (4, 5). Because patients often secrete both monoclonal IgM and abnormal light chains, identifying the pathogenic component can be challenging. A follow-up study conducted by the International Waldenström’s Macroglobulinemia Foundation reported a cumulative incidence of WM-related nephropathy of approximately 5. 1%. The actual incidence may be underestimated. Patients with concurrent renal amyloidosis have a three-fold higher risk of progressing to end-stage renal disease, and their five-year survival rate is only 30–40% (6). Such complications frequently manifest as nephrotic syndrome initially, making them easily confused with common renal disorders. Disease progression is typically rapid, with elderly patients having a particularly poor prognosis (7).

This paper reports the diagnostic and therapeutic process of a patient with WM complicated by AL *λ* − *type* amyloid nephropathy, whose renal involvement was the initial clinical presentation. The analysis focuses on demonstrating that amyloidosis was mediated by *λ* light chain rather than IgM. Additionally, it reviews relevant literature and discusses clinical characteristics of multiple organ involvement to provide guidance for similar clinical scenarios.

## 2 Case report

### 2.1 General information

A 75-year-old female patient was admitted to the Department of Nephrology on May 15, 2023, with facial and bilateral lower-limb edema of 2 days’ duration. The edema was symmetrical and pitting in nature, extending to the knees, and was more pronounced around the eyelids in the morning. The patient also reported persistent foamy urine accompanied by increased urinary frequency and urgency (8–10 times during the day and 3–4 times at night). She denied gross hematuria, oliguria, fever, or other systemic symptoms.

The patient had a history of hypertension for more than 10 years, with previously recorded blood pressure up to approximately 160/90 mmHg. Blood pressure monitoring had been irregular, and long-term standardized antihypertensive treatment had not been maintained. She had been diagnosed with coronary atherosclerotic heart disease 5 years earlier and experienced intermittent chest tightness and exertional shortness of breath during daily activities, which were relieved by rest. No regular secondary prevention therapy had been undertaken. She reported recurrent urinary frequency suggestive of prior urinary tract infection but had not undergone systematic evaluation or treatment. She denied a history of diabetes mellitus, chronic kidney disease, autoimmune disease, malignancy, major surgery, or previous exposure to immunosuppressive or cytotoxic therapy. She had no history of smoking or alcohol consumption and reported no other unhealthy lifestyle habits.

There was no known family history of renal disease, hematological malignancy, amyloidosis, or hereditary disorders. No prior disease-specific interventions had been performed before the current hospitalization.

### 2.2 Physical examination

Body temperature was 36.3 °C, pulse 120 bpm, respiratory rate 20 breaths/min, blood pressure 127/74 mmHg, and BMI 24.0 kg/m^2^. The patient was conscious and cooperative during examination. Mild eyelid edema was observed. Superficial lymph nodes were not palpable, sclerae were not icteric, and mucous membranes showed no cyanosis. The neck was supple, without thyroid enlargement. Breath sounds were clear bilaterally; heart rhythm was regular without pathological murmurs. The abdomen was flat, soft, without palpable liver or spleen enlargement, and negative for shifting dullness. No tenderness was noted in the renal area. Moderate pitting edema of both lower limbs extended to the knees, without skin abnormalities. Muscle strength in all limbs was grade V, physiological reflexes were present, and pathological reflexes were negative.

### 2.3 Auxiliary examinations

#### 2.3.1 Renal system examinations

*Routine urinalysis*: Urine protein 3+, occult blood 2+; urine sediment microscopy revealed red blood cells 5-8/HPF (reference: <3/HPF), white blood cells 2-3/HPF (reference: <5/HPF).

Special urine tests (May 14, 2023): Urine protein 2+, occult blood 2+, urine osmolarity 550 mOsm/kg·H₂O (lower limit of normal), urine pH 6.5.

*Urine protein assessments*: 24-h total protein 6.11 g (reference: <0.15 g), urinary microalbumin 4.89 g/24 h (reference: <30 mg/24 h), urine albumin/creatinine ratio 5,800 mg/g (reference: <30 mg/g).

*Renal function tests*: Serum creatinine 129.90 μmol/L (female reference: 44–97 μmol/L), eGFR 45.2 mL/min/1.73 m^2^ (CKD stage 2), blood urea nitrogen 7.8 mmol/L (reference: 3.2–7.1 mmol/L), uric acid 417.37 μmol/L (female reference: 155–357 μmol/L).

#### 2.3.2 Routine blood and biochemical examinations

*Complete blood count*: White blood cells 5.6 × 10^9^/L, neutrophils 62.3%, lymphocytes 31.5%, hemoglobin 132 g/L, platelets 215 × 10^9^/L, no abnormalities detected.

*Serum biochemistry*: Serum albumin 20.07 g/L (reference: 35–55 g/L), albumin-globulin ratio 0.62 (reference: 1.2–2.4); total cholesterol 6.8 mmol/L (reference: <5.2 mmol/L), triglycerides 2.3 mmol/L (reference: <1.7 mmol/L); liver function and electrolytes were within normal limits.

#### 2.3.3 Myocardial markers and autoantibody examinations

*Myocardial markers*: Elevated NT-proBNP (2877.00 pg./mL; reference <125 pg./mL), mildly increased hs-cTnI (0.0550 ng/mL; reference <0.04 ng/mL), whereas CK-MB remained within the normal range (2.3 ng/mL; reference <4.3 ng/mL).

*Autoantibody profile*: ANA positive (titer 1:100, nuclear granular pattern), β2-glycoprotein 1 antibody-IgM 44.04 RU/ml (reference: <20 RU/ml), erythrocyte sedimentation rate 25 mm/h (female reference: <20 mm/h); specific autoimmune disease antibodies were negative.

#### 2.3.4 Immunoglobulin and light chain examinations

*Serum immunoglobulins*: IgM 28.6 g/L (reference: 0.6–2.6 g/L); IgG and IgA were normal.

*Serum free light chains*: *λ* light chain 63.29 mg/L (reference: 3.3–19.4 mg/L), *κ* light chain 18.49 mg/L (reference: 5.7–26.3 mg/L), *κ*/*λ* ratio 0.2921.

*Urinary free light chains*: *λ* light chain 106.22 mg/L (reference: <0.25 mg/L), κ light chain 37.25 mg/L (reference: <0.13 mg/L), κ/λ ratio 0.3497.

#### 2.3.5 Immunofixation electrophoresis

Monoclonal IgM-*λ* bands were detected by immunofixation electrophoresis in both serum and urine. Urinary Bence-Jones protein was positive *λ* − *type*.

#### 2.3.6 Bone marrow examination

*Bone marrow aspiration and biopsy*: Bone marrow showed hypoplasia (10–20% cellularity); lymphocytes accounted for 32%, with some exhibiting lymphoplasmacytic morphology; plasma cells (3%) displayed normal morphology; Congo red staining was negative; reticulin fiber staining was graded MF-1.

*Flow cytometry*: Mature clonal B lymphocytes (CD5 + CD10- phenotype) accounted for 22.1%, consistent with lymphoplasmacytic proliferative diseases.

*Genetic testing*: MYD88-L265P mutation positive (mutation rate 28.5%); mutations in CXCR4, TP53, and other genes were negative.

#### 2.3.7 Cardiac examination

*Echocardiography (May 17, 2023)*: Left ventricular end-diastolic diameter 54 mm (upper limit of normal), segmental wall-motion abnormalities, mild-to-moderate mitral regurgitation, LVEF 45%.

*Cardiac MRI (May 18, 2023)*: Slight left ventricular enlargement, LVEF 37.67%, reduced local wall motion in the apical segment of the anterior wall.

*Electrocardiogram (July 8, 2023)*: sinus rhythm, normal electrical axis, electrocardiographic abnormalities including QTc prolongation and ST-T changes.

#### 2.3.8 Abdominal ultrasound examination (May 17, 2023)

Ultrasound showed mild fatty liver. Both kidneys were normal in size and shape, with diffusely enhanced renal parenchymal echoes and unclear cortical-medullary boundaries, suggesting renal amyloidosis.

#### 2.3.9 Renal biopsy pathology

**Figure 1:**
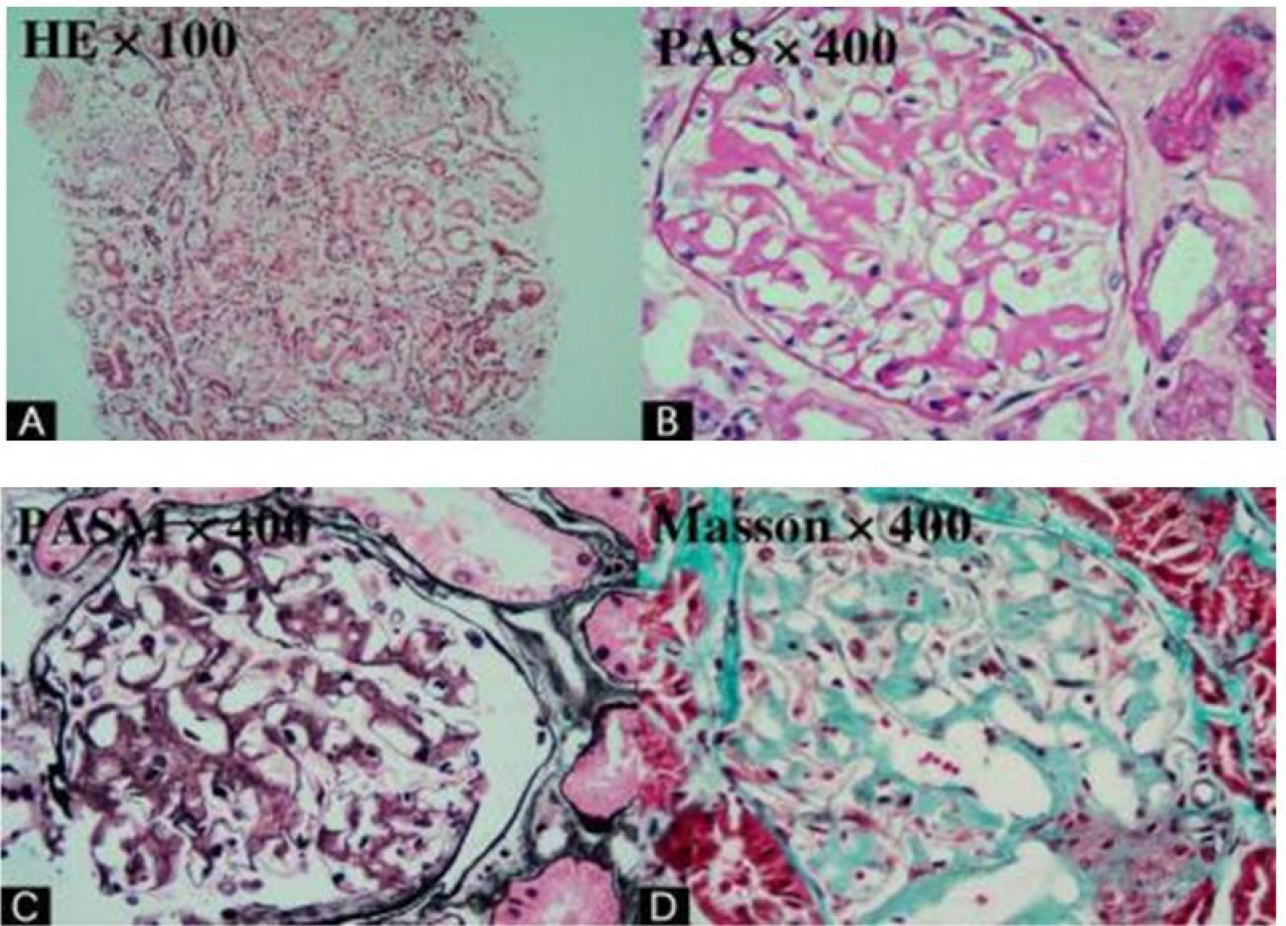
Under light microscopy, 20 glomeruli were observed, 12 of which showed globalsclerosis. **(A,B)** Focal and patchy inflammatory cell infiltration with interstitial fibrosis is observed in the renal interstitium. Eosinophilic proteinaceous deposits are present within the interstitium and the walls of small arteries, accompanied by arterial wall thickening and luminal narrowing. **(C,D)** Abundant amorphous eosinophilic material is deposited in the glomerular mesangium, resulting in mesangial expansion. Segmental irregular thickening of the glomerular basement membrane with eyelash-like projections is also noted.

*Light microscopy*: Among 20 glomeruli, 12 showed global sclerosis (60%); the remaining 8 glomeruli had significant widening of mesangial areas, with abundant deposition of pink, homogeneous, acellular proteinaceous material in mesangial regions and capillary loops. Deposits were strongly PAS-positive. PASM staining revealed segmental basement membrane thickening, with irregular “eyelash-like” protrusions on the outer basement membrane. Approximately 40% of renal tubules exhibited focal atrophy, vacuolar degeneration of tubular epithelial cells, dilation of lumens, and protein casts. Interstitial tissue showed diffuse inflammatory cell infiltration (mainly lymphocytes and monocytes) with moderate fibrosis (about 30%). Small arterial walls exhibited thickening, hyalinization, and proteinaceous deposits (Figure 1). Congo red staining was positive (deposited extracellular material was red), oxidized Congo red staining was positive, and apple-green birefringence appeared under polarized light microscopy, confirming amyloid deposits.

**Figure 2:**
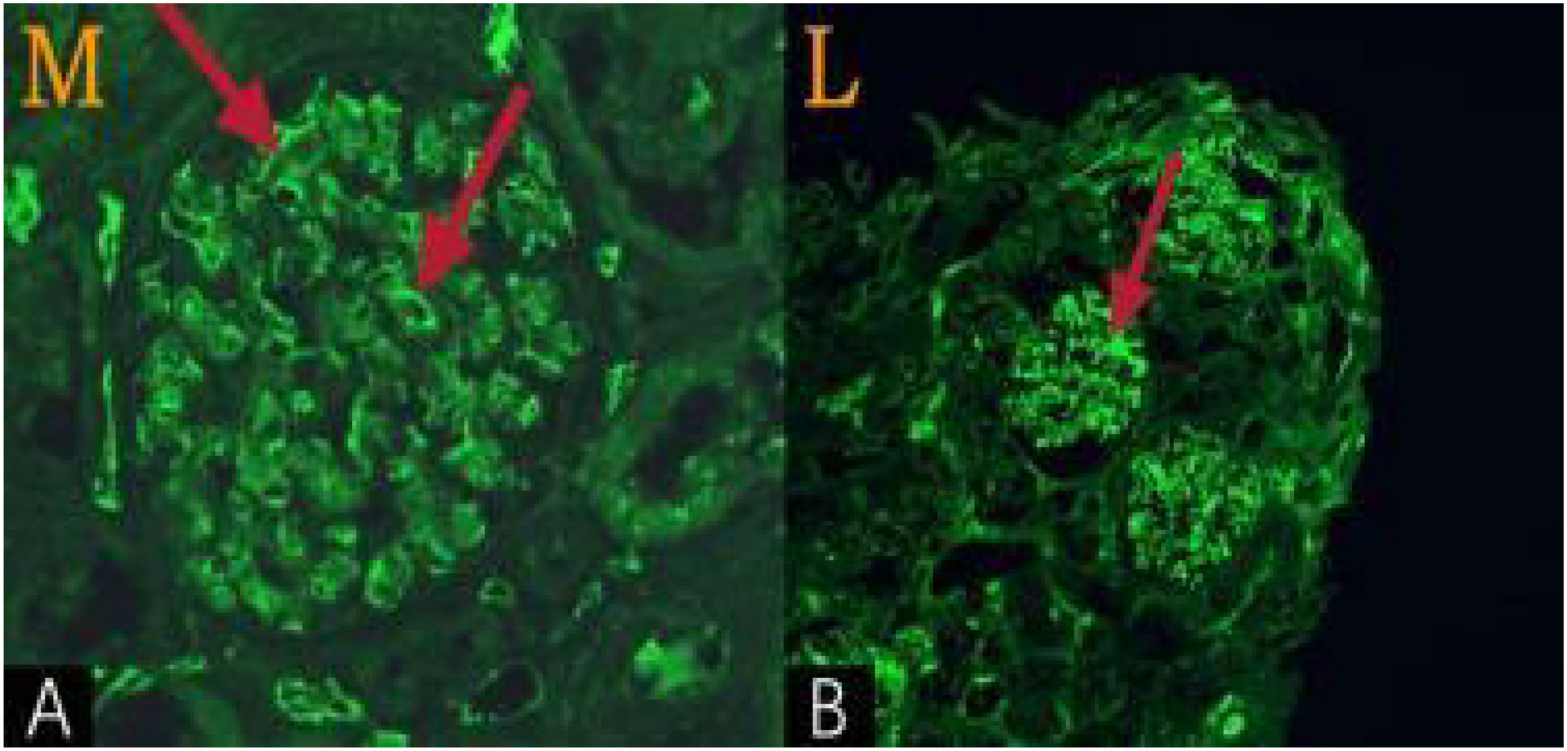
Immunofluorescence examination **(A)** showed positive staining for IgM, with linear enhancement of fluorescence around the glomerular capillary walls, indicating that IgM was concentrated in the glomerular capillary walls. **(B)** Showed positive staining for light chain L, with irregular patchy enhancement of fluorescence, suggesting that light chain L was mainly deposited in the mesangial area.

*Immunofluorescence examination*: Both IgM and *λ* light chains were positive in serum and urine immunofixation electrophoresis. To clarify which component was pathogenic, the immunofluorescence protocol was optimized, using indirect immunofluorescence to identify deposition sites and distribution characteristics. *λ* light chains showed diffuse, granular deposition (+) predominantly in the glomerular mesangial area, basement membrane, and renal interstitial vessel walls. IgM demonstrated weak (+/−) and scattered positivity, limited to capillary walls, without specific deposition in the mesangium (Figure 2). *κ* light chains, IgG, IgA, C3, C4, C1q, and Fib were negative, confirming deposition restricted to a single light chain. The pathological mechanism of amyloidosis is closely related to selective deposition of pathogenic proteins in specific organ sites, with the mesangium as the typical site of renal amyloidosis. This difference in deposition location was crucial for identifying pathogenic components. Mass spectrometry or co-localization analysis remains the gold standard for determining pathogenic proteins in amyloidosis, directly quantifying protein deposition ratios and fiber composition. However, these methods could not be performed due to limited conditions at our center. Therefore, an alternative method based on the “matching of deposition sites with typical disease features,” along with clinical and pathological findings, was adopted for comprehensive diagnosis confirmation. Differentiation from WM complicated with light chain deposition disease (LCDD) is necessary. LCDD exhibits electron-dense deposits under electron microscopy, with light chains mainly deposited in the glomerular basement membrane. This differs from the current case, which lacked electron-dense deposits ultrastructurally and featured mesangial deposition. A previous WM case complicated by AL-type renal amyloidosis [Abstract 2] was distinguished from LCDD based on electron microscopic fibril diameters (8.32 ~ 9.56 nm). The fibril diameter in this case was 8–12 nm, consistent with AL-type amyloidosis.

**Figure 3:**
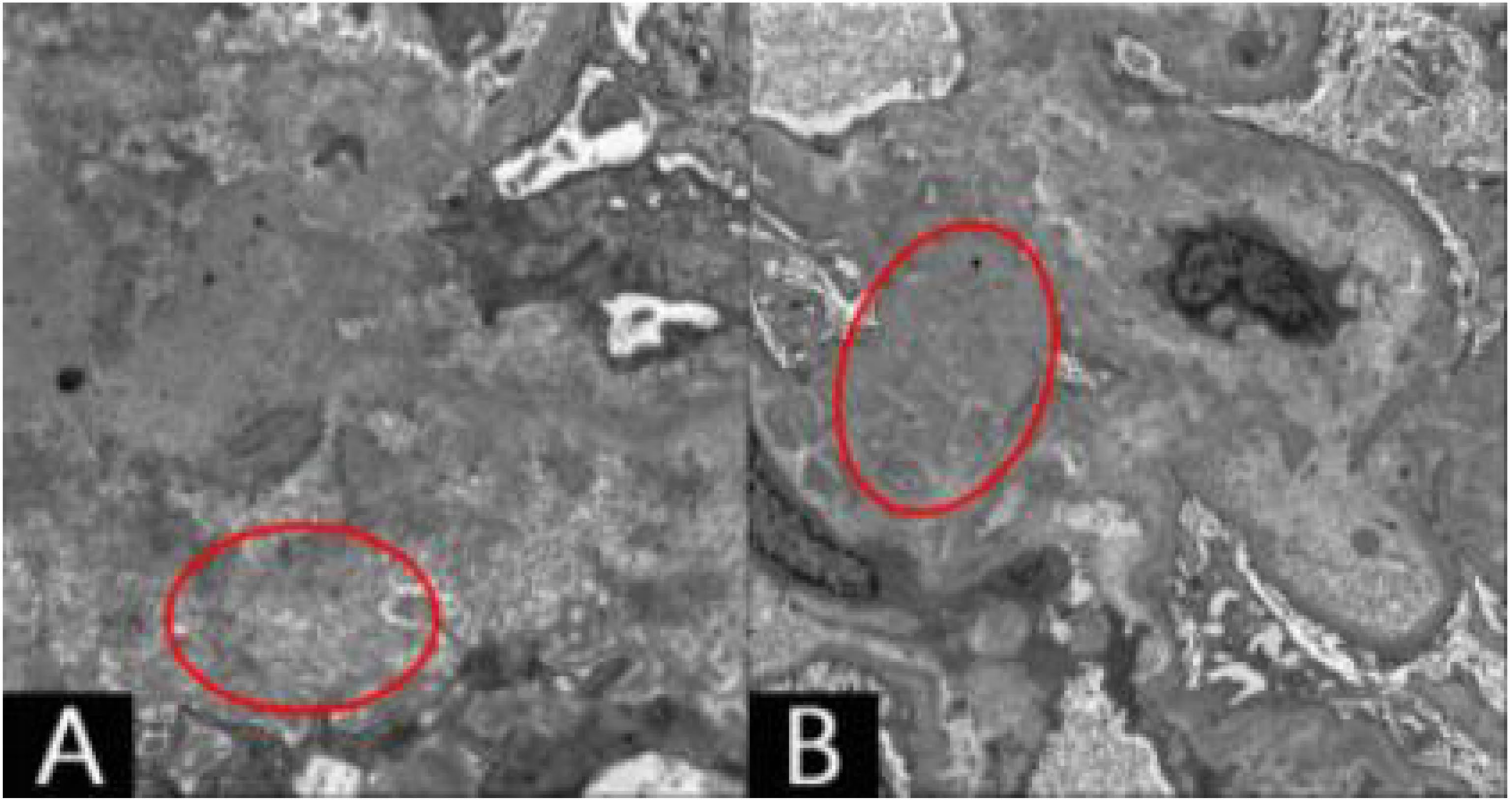
Under electron microscopy **(A,B)** extensive deposition of fibrillary material can be seen within the red circles, which are approximately 8–12 nm in diameter, rigid, unbranched, and disorderly arranged.

*Electron microscopy*: Transmission electron microscopy showed segmental thickening of the glomerular basement membrane (250–600 nm), diffuse fusion of podocyte foot processes (approximately 80% fusion), and no electron-dense deposits. Numerous fibrillar deposits (8–12 nm diameter), rigid, unbranched, and randomly arranged, appeared in the glomerular mesangium, renal interstitium, and small arterial walls, consistent with amyloid fibril ultrastructure (Figure 3). Cytoplasm of renal tubular epithelial cells exhibited occasional vacuoles; mitochondria were not notably swollen. Renal interstitial capillaries appeared normal, and no viral particles or pathogens were observed (Table 1).

**Table 1:** Timeline of clinical course.

Time	Event description	Key findings	Treatment/outcome
May 2023 (2 days before admission)	Symptoms onset: facial swelling and bilateral lower-limb edema, urinary frequency and urgency	Progressive fluid retention	Patient sought medical attention
May 14 2023	Pre-admission laboratory examination	Proteinuria and microscopic hematuria detected	Subsequent hospital admission
May 15 2023	Hospitalization for edema and fatigue	Nephrotic syndrome with renal impairment, hypoalbuminemia, IgM-l protein with abnormal free light-chain profile, elevated myocardial biomarkers	Comprehensive diagnostic evaluation initiated
May 17–212,023	Comprehensive evaluation for nephrotic syndrome and suspected systemic disease	Abdominal ultrasound suggested renal amyloidosis; echocardiography showed reduced LVEF; cardiac MRI showed reduced LVEF; immunofixation revealed IgM- l monoclonal protein; bone marrow examination showed lymphoplasmacytic infiltration and MYD88 mutation; renal biopsy confirmed AL amyloid deposition	Final diagnosis of Waldenström macroglobulinemia with AL l - type amyloid nephropathy and cardiac involvement
May 22 2023	Initiation of systemic therapy		RCD chemotherapy started
≈ 1 week after first cycle	Symptomatic improvement in edema and foamy urine	Reduction in proteinuria and serum free light-chain level with improvement in renal function	Early treatment response observed
July 8 2023	Cardiac reassessment during chemotherapy	Electrocardiography showed sinus rhythm with a normal electrical axis, QTc prolongation, and ST-T segment changes	Continued RCD chemotherapy with close cardiac monitoring
After four cycles (≈ Aug 2023)	Marked clinical improvement	Minimal residual proteinuria with recovery of renal and cardiac function	Partial remission achieved
Oct 20 2023	Clinically stable condition at discharge	Laboratory indicators improved	Patient discharged with follow-up plan
1 month after discharge	No obvious discomfort	No significant laboratory deterioration	Continued outpatient management
2 months after discharge	Fatigue, chest tightness, shortness of breath, and lower-limb edema	Increased proteinuria and serum creatinine with light chain rebound and decline in cardiac function	Disease relapse; patient declined further chemotherapy
3 months after discharge	Rapid clinical deterioration with generalized edema, fever, and oliguria	Severe renal dysfunction with worsening cardiac function and pulmonary infection	Emergency admission to intensive care unit
Jan 28 2024	Progression to multiple organ failure	Combined cardiac, renal, and respiratory failure	Treatment withdrawn; patient died

### 2.4 Diagnosis and treatment

Final diagnosis:

(1) WM;
(2) AL *λ* − *type* amyloid nephropathy mediated by λ light chains;
(3) Nephrotic syndrome;
(4) Chronic kidney disease stage 2 (CKD stage 2);
(5) Cardiac amyloidosis (reduced left ventricular systolic function).

### 2.5 Treatment regimen

After informed consent was obtained from the patient and her family, RCD chemotherapy was initiated on May 22, 2023. Each cycle lasted 21 days, for a total of four cycles, and was combined with supportive symptomatic treatment.

*Chemotherapy regimen*: Rituximab 544 mg (375 *mg*/*m*^2^) and cyclophosphamide 1,088 mg (750 *mg*/*m*^2^) were administered intravenously on day 1. Dexamethasone 40 mg was given orally on days 1–4. Chemotherapy was suspended on days 5–21, during which supportive therapy was maintained. This regimen is a category 2A recommendation for elderly patients with WM, with low toxicity and a therapeutic focus on suppressing *λ* light chain production and limiting target organ damage (3).

*Supportive treatment*: Included gastric protection (pantoprazole), albumin supplementation, infection prophylaxis (piperacillin-tazobactam and acyclovir), diuretic therapy (furosemide, spironolactone, and hydrochlorothiazide), renal-protective therapy including traditional (Chinese medicines such as Shenshuaining and Jinshuibao), electrolyte correction (potassium chloride), osteoporosis prevention (Caltrate D and calcitriol), and cardiac protection (metoprolol and spironolactone). Clinical parameters were closely monitored throughout treatment.

### 2.6 Efficacy evaluation during treatment

One week after completion of the first cycle, edema and foamy urine were alleviated. Serum creatinine decreased to 105 μmol/L, serum albumin increased to 25.3 g/L, 24-h urinary protein decreased to 3.2 g, and serum free *λ* light chain decreased to 38.5 mg/L. Only mild leukopenia occurred and resolved after symptomatic treatment, indicating good treatment tolerance. After four cycles, serum immunofixation electrophoresis became negative, and the proportion of bone marrow clonal B lymphocytes decreased to 8.2%. Renal function returned to normal (serum creatinine 89 μmol/L, eGFR 62.5 mL/min·1.73 m^2^). Serum free *λ* light chain decreased to 21.3 mg/L, with a *κ*/λ ratio of 0.48, and IgM decreased to 22.7 g/L. The 24-h urinary protein decreased to 0.52 g, and serum albumin increased to 38.6 g/L. Echocardiography showed improved cardiac function, with LVEF increasing to 42.3%, mitral regurgitation reduced to trace, NT-proBNP decreased to 850 pg./mL, and hs-cTnI returned to normal, confirming that the treatment regimen was effective.

### 2.7 Disease outcome

The patient was discharged on October 20, 2023, with instructions to continue oral medications, follow a low-salt, low-fat, high-quality protein diet, avoid fatigue and nephrotoxic medications, and undergo regular follow-up examinations.

*One month post-discharge*: All indicators remained stable, without discomfort;

*Two months post-discharge*: Fatigue, chest tightness, and shortness of breath worsened. Bilateral lower extremity edema recurred. The 24-h urinary total protein increased to 3.8 g, serum albumin decreased to 28.3 g/L, serum creatinine rose to 138 μmol/L, BNP elevated to 4,260 pg./mL, serum free *λ* light chain rebounded to 41.5 mg/L, and LVEF decreased to 35.2%. The patient refused further chemotherapy;

*Three months post-discharge*: The disease rapidly progressed, with severe generalized edema, fever, and oliguria. The 24-h urinary total protein reached 8.7 g, serum albumin dropped to 18.9 g/L, serum creatinine elevated to 267 μmol/L, corresponding to stage-4 chronic kidney disease (CKD stage 4). The condition was further complicated by pulmonary infection and progressive cardiac dysfunction (LVEF 28.7%). The patient was admitted to the ICU for emergency management. Due to multiple organ failure involving the heart, kidneys, and respiratory system, the family discontinued treatment. The patient died on Jan 28, 2024, shortly after voluntarily leaving the hospital.

## 3 Discussion

### 3.1 Key diagnostic and differential diagnostic points

The diagnosis of WM requires clonal lymphoplasmacytic infiltration of the bone marrow (≥10%) and the presence of monoclonal IgM paraprotein (8). In this case, serum and urine immunofixation electrophoresis demonstrated monoclonal IgM-*λ* immunoglobulin, bone marrow showed 32% lymphoplasmacytic infiltration, flow cytometry and MYD88-L265P mutation were positive, thus meeting WM diagnostic criteria (3). The mutation rate of MYD88-L265P is extremely low in similar diseases, making it a critical differential diagnostic marker (9).

The diagnosis of renal AL amyloidosis depends on renal biopsy (2, 3). Typical features include ultrastructural amyloid fibrils of 7–10 nm, restricted deposition of a single light chain, and positive Congo red staining (4). In this case, immunofluorescence revealed core deposition of *λ* light chains in the mesangium, whereas IgM deposition was limited to capillary walls. Electron microscopy and Congo red staining confirmed the amyloid characteristics, establishing the diagnosis of AL *λ* − *type* amyloid nephropathy (1, 4).

In this patient, careful differential diagnosis was necessary to distinguish Waldenström macroglobulinemia with AL amyloidosis from other IgM-related disorders, including IgM-MGUS, MGRS, IgM multiple myeloma, ATTR amyloidosis, and hepatic amyloidosis. 264 Specifically: ① IgM-MGUS presents no organ damage and <10% clonal cell infiltration; ② MGRS lacks hematological tumor pathology and molecular markers, often showing complement activation; ③ IgM-MM features osteolytic lesions and CD138 + plasma cell proliferation; ④ ATTR amyloidosis shows negative light chain staining, often accompanied by myocardial hypertrophy; ⑤ Hepatic amyloidosis is typically associated with markedly elevated alkaline phosphatase levels (3). The present case clearly excluded these conditions based on clinical characteristics.

Cardiac examination revealed decreased LVEF and segmental wall motion abnormalities. Combined with elevated monoclonal light chains and renal biopsy results, cardiac amyloidosis was confirmed, preventing misdiagnosis as ischemic cardiomyopathy. The ultrasound finding of “diffusely enhanced renal parenchymal echo” also provides diagnostic reference for primary hospitals.

### 3.2 Core innovation: key evidence that *λ* light chain, rather than IgM, mediates amyloidosis

In WM complicated by amyloidosis, accurate identification of pathogenic components is essential for treatment. In this case, multidimensional evidence confirmed that *λ* light chain was the sole pathogenic component:

#### 3.2.1 Pathological evidence

*λ* light chains exhibited core mesangial deposition, consistent with AL- type amyloidosis. IgM lacked targeted mesangial deposition, and electron microscopy showed no immune complex-like electron-dense deposits.

#### 3.2.2 Correlation with clinical indicators

*λ* light chain levels closely correlated with cardiorenal function impairment. Reductions in λ light chain after treatment matched symptom improvement, while elevations corresponded to disease progression. In contrast, IgM fluctuations showed no clear relationship with disease severity.

#### 3.2.3 Molecular mechanism

*λ* light chains, due to their small molecular weight, easily misfold into amyloid fibrils. IgM, a stable pentameric structure, rarely forms fibrils, making IgM-mediated amyloidosis clinically uncommon (5).

#### 3.2.4 Therapeutic verification

The RCD regimen significantly reduced *λ* light chain secretion and achieved remarkable clinical effects, further confirming the central pathogenic role of λ light chains.

### 3.3 Selection of treatment strategies

The core therapeutic strategy for WM complicated by AL amyloidosis is inhibition of abnormal light chain production. Treatment regimens should be tailored according to patient age, tolerance, and pathogenic components (7). The patient in this case was 75 years old and had existing cardiorenal impairment; thus, the RCD chemotherapy regimen was selected. This regimen has low toxicity, is suitable for elderly patients, and aligns with guideline recommendations (3). After treatment, the patient’s *λ* light chain levels decreased and cardiorenal function improved, confirming regimen effectiveness (3, 5, 7).

Autologous stem cell transplantation can be considered for younger patients in good physical condition but is unsuitable for elderly patients or those with significant organ damage (10). BTK inhibitors are highly effective (response rate 80–90%) for WM with positive MYD88 mutation, and can serve as second-line therapy for patients experiencing light chain rebound post-chemotherapy (6, 11).

### 3.4 Prognostic analysis and clinical implications

Despite achieving short-term remission after RCD chemotherapy, the patient experienced rapid disease progression and ultimately had an unfavorable outcome. Several factors likely contributed to the poor prognosis. First, extensive *λ* light-chain deposition had already resulted in irreversible structural damage to the kidneys and myocardium at the time of diagnosis. Second, cardiac involvement manifested by reduced left ventricular ejection fraction indicated advanced systemic amyloidosis and was associated with increased mortality risk. In addition, the aggressive biological behavior related to the MYD88-L265P mutation and the limited tolerance for intensive therapy in an elderly patient may have further restricted therapeutic options. Notably, biochemical relapse characterized by a rebound in serum free *λ* light-chain levels was not followed by timely therapeutic escalation because the patient declined further chemotherapy. Persistent production of amyloidogenic light chains may have accelerated progressive multi-organ dysfunction, thereby contributing to rapid clinical deterioration.

This case highlights several important clinical implications. Early recognition of Waldenström macroglobulinemia complicated by AL amyloidosis is essential in elderly patients presenting with nephrotic syndrome and monoclonal IgM. Multimodal diagnostic evaluation, including renal biopsy and bone marrow examination, remains crucial for accurate diagnosis. Furthermore, close longitudinal monitoring of serum free light-chain levels and cardiorenal function is necessary to detect early relapse and guide timely therapeutic adjustment. Finally, the integration of novel targeted therapies may represent a promising direction for improving outcomes in high-risk patients.

### 3.5 Strengths and limitations

This case has several strengths. The diagnosis was established using a comprehensive multimodal approach including renal biopsy, bone marrow examination, immunofixation electrophoresis, genetic testing, and dynamic monitoring of serum free *λ* light chains, all of which are essential for accurate disease evaluation in Waldenström macroglobulinemia (5, 12). Renal biopsy remains the foundation for diagnosis of renal amyloidosis, with Congo red staining and ultrastructural examination providing definitive evidence of amyloid deposition (13, 14). In the present case, the diagnostic findings were further supported by structured longitudinal follow-up including serial assessment of nephrotic syndrome, serum free *λ* light chain level, renal function parameters, and cardiac function (8, 12). Such multidimensional monitoring allowed real-time evaluation of therapeutic efficacy and facilitated early identification of biochemical relapse, thereby contributing to a more comprehensive understanding of disease progression (3, 5, 6).

However, several limitations should be acknowledged. Advanced techniques such as laser microdissection combined with mass spectrometry are currently regarded as gold standard for precise amyloid typing (15), were not available at our institution. Therefore, identification of the pathogenic *λ* light chain was primarily based on indirect immunofluorescence findings together with clinicopathological correlation (14, 16). In addition, although renal biopsy findings were consistent with AL amyloidosis, further evaluation for systemic autoimmune renal diseases such as testing for anti-double stranded DNA antibodies, complement levels, and antineutrophil cytoplasmic antibodies was not comprehensively performed (17, 18). Consequently, the possibility of overlapping immune-mediated glomerular pathology including lupus nephritis or cryoglobulinemic glomerulonephritis, cannot be completely excluded. Furthermore, as this report describes the clinical course of a single patient, the findings should be interpreted with caution and may not be directly generalizable to the broader population of patients with Waldenström macroglobulinemia-associated amyloidosis (1, 3, 7, 12). Finally, interruption of further chemotherapy following biochemical relapse limited the opportunity to evaluate alternative therapeutic strategies and long-term outcomes (3, 5, 8, 17).

## 4 Conclusion

This case report analyzes a 75-year-old female diagnosed with Waldenström Macroglobulinemia (WM) complicated by AL (*λ*-type) amyloid nephropathy—enriching clinical insights into this rare comorbidity that accounts for approximately 2% of all WM cases globally. As a subtype of lymphoplasmacytic lymphoma, WM is rarely associated with AL amyloidosis, and the concurrence of both conditions poses unique diagnostic and therapeutic challenges, especially in elderly patients with multiple organ involvement.

The patient presented with classic manifestations of nephrotic syndrome, including facial and bilateral lower-extremity pitting edema, persistent foamy urine, and increased urinary frequency/urgency, alongside laboratory abnormalities: significantly elevated serum IgM (28.6 g/L, far exceeding the normal range of 0.6–2.6 g/L), a marked increase in serum free *λ* light chain (63.29 mg/L) with a reversed *κ*/λ ratio (0.2921), hypoalbuminemia (20.07 g/L), and impaired renal function (serum creatinine 129.90 μmol/L, eGFR 45.2 mL/min·1.73 m^2^). Diagnosis was confirmed through a multi-modality approach: (1) WM was validated by bone marrow biopsy showing 32% clonal lymphoplasmacytic infiltration, positive MYD88-L265P mutation (28.5% mutation frequency), and immunofixation electrophoresis detecting monoclonal IgM-*λ* in both serum and urine; (2) AL (λ-type) amyloidosis was confirmed via renal biopsy, which revealed diffuse λ light chain deposition in the glomerular mesangium, positive Congo red staining with apple-green birefringence under polarized light, and transmission electron microscopy showing 8–12 nm unbranched amyloid fibrils, combined with cardiac evaluations indicating reduced left ventricular ejection fraction (LVEF) consistent with cardiac amyloidosis.

The rituximab-cyclophosphamide-dexamethasone (RCD) regimen—recommended as a Category 2A treatment for elderly WM patients in the 2022 Chinese Expert Consensus on LPL/WM—was administered, achieving notable short-term efficacy. After four 21-day cycles, the patient’s renal function normalized (serum creatinine 89 μmol/L, eGFR 62.5 mL/min·1.73 m^2^), serum free *λ* light chain decreased to 21.3 mg/L, IgM levels declined to 22.7 g/L, 24-h urinary protein dropped to 0.52 g, and cardiorenal symptoms substantially improved. This therapeutic response further validated *λ* light chains as the key driver of amyloidogenesis, supported by pathological evidence of targeted mesangial deposition, clinical correlation between λ light chain fluctuations and disease activity, molecular characteristics favoring light chain misfolding, and direct therapeutic suppression of *λ* light chain secretion.

Despite initial success, the patient experienced rapid disease progression and died on Jan 28, 2024, approximately 3 months after discharge, due to multi-organ failure involving the heart, kidneys, and respiratory system. Keyprognostic risk factors included irreversible organ damage from long-term *λ* light chain deposition (60% of glomeruli showed global sclerosis on renal biopsy), severe cardiac involvement (baseline LVEF < 40%), advanced age (75 years) precluding autologous stem cell transplantation, the aggressive nature of the MYD88-L265P mutation, and delayed intervention following *λ* light chain rebound.

Key clinical implications: (1) For elderly patients presenting with nephrotic syndrome accompanied by abnormal IgM and free light chain levels, prompt bone marrow and renal biopsy are critical to rule out WM-related AL amyloidosis and avoid misdiagnosis; (2) Treatment should prioritize targeting pathogenic *λ* light chains rather than solely focusing on IgM reduction to halt amyloid deposition and organ damage; (3) Bi-monthly follow-up is recommended for high-risk patients to closely monitor λ light chain levels, cardiorenal function, and disease progression, enabling timely adjustment of treatment strategies to improve outcomes.

## Data Availability

The original contributions presented in the study are included in the article/supplementary material, further inquiries can be directed to the corresponding author/s.

## Glossary

AL: Amyloid light chain
ANA: Antinuclear antibody
CK-MB: Creatine kinase-MB
C3: Complement component 3
C4: Complement component 4
C1q: Complement component 1q
CXCR4: C-X-C chemokine receptor type 4
eGFR: Estimated glomerular filtration rate
Fib: Fibrinogen
HPF: High power field
IgA: Immunoglobulin A
IgG: Immunoglobulin G
IgM: Immunoglobulin M
LCDD: Light chain deposition disease
LPL: Lymphoplasmacytic lymphoma
LVEF: Left ventricular ejection fraction
MF-1: Grade 1 Myelofibrosis
MGUS: Monoclonal gammopathy of undertermined significance
MGRS: Monoclonal gammopathy of renal significance
MRI: Magnetic resonance imaging
MYD88: Myeloid differentiation primary response 88
NT-proBNP: N-terminal pro b-type natriuretic peptide
PAS: Periodic acid–Schiff
PASM: Periodic Acid Silver Methenamine
RCD: Rituximab, Cyclophosphamide, and Dexamethasone
TP53: Tumor protein 53
WM: Waldenström macroglobulinemia
